# Transforaminal Epidural Steroid Injection Versus Oral Corticosteroids for Acute Lumbar Radiculopathy: A Prospective Observational Comparative Study

**DOI:** 10.7759/cureus.105529

**Published:** 2026-03-19

**Authors:** Maheshwar Lakkireddy, Syed Ifthekar, Shaikh Mohd ahmed Mohd jaish, Ranjith K Yalamanchili, Srikanth Eppakayala, Deepak Kumar Maley, Deepankar Satapathy

**Affiliations:** 1 Orthopaedics, All India Institute of Medical Sciences, Bibinagar, Hyderabad, IND

**Keywords:** low back pain, lumbar radiculopathy, modified oswestry disability index, oral corticosteroids, transforaminal epidural steroid injection

## Abstract

Introduction: Acute low back pain with radicular symptoms is a common and disabling condition. Corticosteroids are frequently used when conservative management fails; however, the comparative effectiveness of transforaminal epidural steroid injection (TFESI) versus oral corticosteroid therapy remains inadequately defined. This prospective observational comparative study aimed to evaluate and compare short-term pain reduction (Visual Analogue Scale [VAS]), functional improvement (Modified Oswestry Disability Index [MODI]), and neural tension changes (Straight Leg Raise Test [SLRT]) at 12 weeks between TFESI and oral corticosteroid therapy in patients with MRI-confirmed acute lumbar radiculopathy.

Materials and methods: This prospective observational study was conducted at a tertiary care center between June 2023 and December 2024. Treatment allocation was non-randomized and determined as part of routine clinical practice. Thirty-four patients aged 18-70 years with MRI-confirmed lumbar disc herniation and persistent radicular pain after at least three weeks of conservative treatment were enrolled. Patients received either a single fluoroscopy-guided TFESI with 40 mg triamcinolone acetonide (n = 17) or a 15-day tapering course of oral prednisolone (total dose 600 mg; n = 17). Outcomes were assessed at baseline and at 1, 3, 6, and 12 weeks using VAS for back and leg pain, MODI, and SLRT.

Results: Baseline demographic, clinical, and radiological characteristics were comparable between groups. The TFESI group demonstrated significantly greater improvement in VAS back pain, VAS leg pain, MODI scores, and SLRT values at 1, 3, and 6 weeks compared with the oral steroid group (p < 0.001). Early median reductions in leg pain (70%) and back pain (60%) and marked functional improvement were observed following TFESI. By 12 weeks, between-group differences diminished and were no longer statistically significant, indicating convergence of outcomes over time.

Conclusion: In this prospective non-randomized cohort, TFESI was associated with faster and more pronounced short-term pain relief and functional improvement than oral corticosteroids in patients with acute lumbar radiculopathy; however, outcomes were comparable by 12 weeks.

## Introduction

Low back pain (LBP) is one of the most prevalent musculoskeletal disorders worldwide and remains a leading cause of disability across all age groups [1]. Nearly 80% of adults experience at least one episode of low back pain during their lifetime, imposing a substantial burden on healthcare systems and society [2]. Among the various clinical presentations of LBP, radicular pain characterized by pain radiating along the distribution of a spinal nerve results in greater pain severity, functional limitation, and reduced quality of life [3]. Lumbar radiculopathy most commonly arises from intervertebral disc herniation, foraminal stenosis, or degenerative spinal changes leading to nerve root compression and inflammation [4].

The pathophysiology of lumbar radiculopathy involves a complex interplay of mechanical and inflammatory mechanisms [5]. The herniation of disc material causes direct mechanical compression of the nerve root while simultaneously triggering an inflammatory cascade through the release of phospholipase A2, cytokines, prostaglandins, and other inflammatory mediators from the nucleus pulposus [6]. This inflammatory milieu sensitizes nerve roots and perpetuates pain. Consequently, corticosteroids, owing to their potent anti-inflammatory effects, have been widely employed to attenuate nerve root inflammation and alleviate radicular symptoms [7].

Current clinical guidelines advocate initial conservative management for acute lumbar radiculopathy, including analgesics, physical therapy, and activity modification, typically for a duration of 4-6 weeks [8]. While most patients improve with these measures, a subset continues to experience significant pain and disability, necessitating escalation of treatment. Corticosteroids may be administered either orally or via epidural injection. Oral corticosteroids are non-invasive, inexpensive, and easily accessible, but their efficacy has been inconsistent across studies, and concerns remain regarding systemic adverse effects [9]. Epidural steroid injections, particularly transforaminal epidural steroid injections (TFESIs), allow targeted delivery of corticosteroids to the anterior epidural space adjacent to the affected nerve root, potentially enhancing therapeutic efficacy while limiting systemic exposure [10].

Despite widespread clinical use of both modalities, direct comparative evidence between TFESIs and oral corticosteroids for acute lumbar radiculopathy remains limited. Available studies are often constrained by small sample sizes, heterogeneous patient populations, and variable outcome measures [11-14]. In this context, establishing whether oral corticosteroids provide outcomes comparable to TFESIs could have significant implications for clinical decision-making.

Therefore, this prospective observational comparative study was designed to evaluate and compare short-term pain reduction (Visual Analogue Scale [VAS]), functional improvement (Modified Oswestry Disability Index [MODI]), and neural tension changes (Straight Leg Raise Test [SLRT]) over a 12-week follow-up period between patients receiving TFESI and those receiving oral corticosteroid therapy for MRI-confirmed acute lumbar radiculopathy.

## Materials and methods

**Figure 1:**
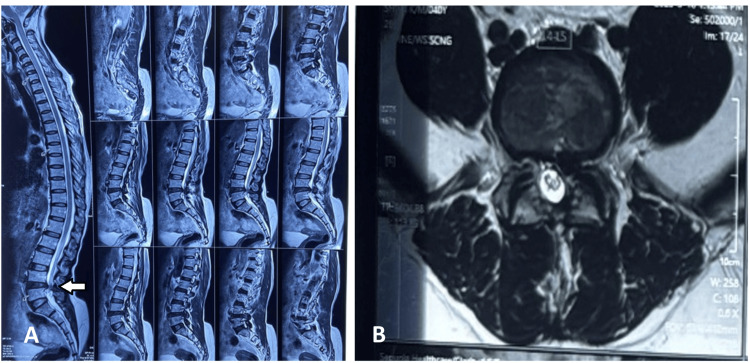
MRI lumbar spine showing prolapsed intervertebral disc at L4–L5 level (A) Sagittal T2-weighted magnetic resonance imaging of the lumbosacral spine demonstrating a posterior disc protrusion at the L4–L5 intervertebral level, indenting the thecal sac and causing anterior compression. Reduced disc height and degenerative signal changes are also noted at the affected level. (B) Axial T2-weighted image at the L4–L5 level showing a left paracentral disc bulge/protrusion causing compression of the traversing left L5 nerve root within the lateral recess, with associated narrowing of the spinal canal.

This prospective observational study was conducted in the Department of Orthopaedics, All India Institute of Medical Sciences (AIIMS), Bibinagar, from June 2023 to December 2024. The study design was non-randomized, and no randomization sequence was generated. Treatment allocation was determined as part of routine clinical practice by the treating orthopedic consultant in consultation with the patient. The study included male and female patients aged 18-70 years presenting with acute low back pain associated with radicular symptoms. Radiculopathy was defined by the presence of at least one of the following clinical features: dermatomal leg pain, myotomal weakness, or dermatomal sensory deficit, with clinical findings correlating with magnetic resonance imaging (MRI) evidence of lumbar disc herniation causing nerve root compression [15]. Patients with radicular low back pain persisting for more than three weeks and within 12 weeks of symptom onset were included, consistent with the acute phase of lumbar radiculopathy (Figure 1).

Patients presenting to the orthopedic outpatient department with radicular low back pain persisting for more than three weeks were clinically evaluated, including assessment using the Straight Leg Raise Test (SLRT). Patients with a positive SLRT (<70°) underwent an MRI of the lumbosacral spine. Those with MRI-confirmed lumbar disc herniation at L4-L5 or L5-S1 levels (single-level or two-level involvement with identification of a clinically dominant symptomatic level) with concordant clinical findings were screened using predefined eligibility criteria and enrolled after obtaining written informed consent. Participants were enrolled consecutively and followed prospectively after initiation of treatment selected by the treating clinician.

Patients aged 18-70 years with radiculopathy not relieved after at least three weeks of conservative treatment were included if they had MRI-proven lumbar disc herniation with clinical symptoms correlating with imaging findings, a Visual Analogue Scale (VAS) score greater than 5, leg pain severity exceeding back pain severity on VAS, and a Modified Oswestry Disability Index (MODI) score greater than 40%. Conservative treatment prior to enrollment consisted of activity modification, oral non-steroidal anti-inflammatory drugs (NSAIDs), and supervised home-based back-strengthening exercises. Patients were excluded if they were on anticoagulation therapy or had bleeding disorders; presented with cauda equina syndrome; had hypersensitivity to the injected substances, active systemic or local infection, uncontrolled diabetes mellitus or congestive heart failure, spinal deformity, prior spinal surgery, or previous epidural steroid injection; had malignancy-related low back pain; or had a history of gastrointestinal bleeding, uncontrolled hypertension, psychosis, or immunosuppression.

Convenience sampling was employed. No attempt was made by the investigators to influence treatment allocation.

Sample size estimation was performed using OpenEpi software (Version 3.01) for comparison of means between two independent groups using the formula:

𝑛 = (𝑍𝛼/2 + 𝑍𝛽)² (𝜎₁² + 𝜎₂²) / (𝜇₁ − 𝜇₂)²,

where Zα/2 = 1.96 (95% confidence interval), Zβ = 0.84 (80% power), σ1 = 1.9, σ2 = 2.2, μ1 = 5.5, and μ2 = 3.2. The calculated sample size was 13 participants per group. After accounting for an anticipated dropout rate of 15%, the final sample size was adjusted to 17 participants per group, resulting in a total of 34 patients. The assumed mean difference (μ1 − μ2 = 2.3) was derived from previously published VAS reduction data and exceeded the established minimum clinically important difference (MCID) for radicular pain, thereby supporting clinical relevance. The estimation was guided by effect sizes reported in previous studies by Ko et al. and Imami et al. [9,16].

**Figure 2:**
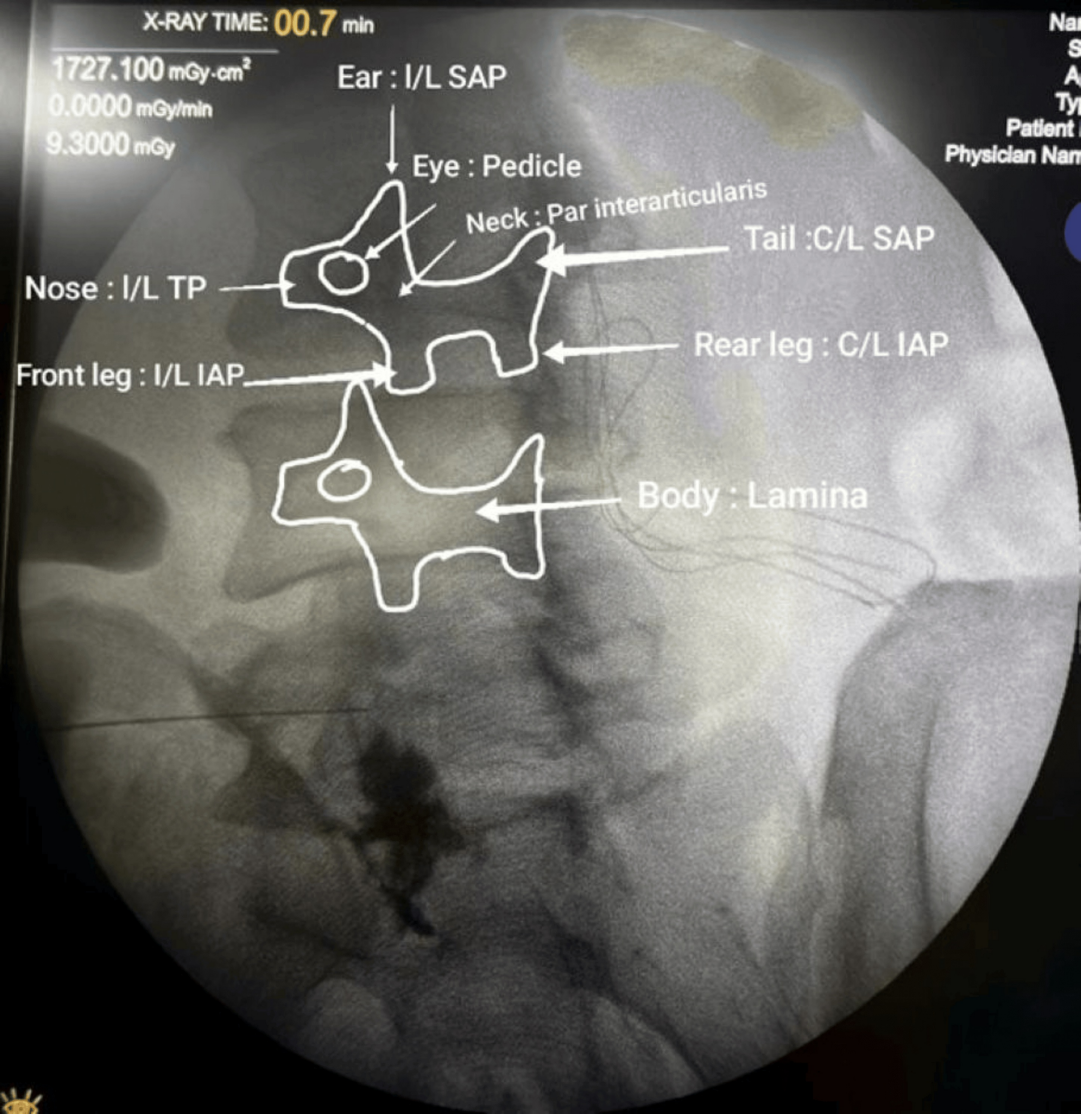
Scottie dog appearance Under fluoroscopic guidance, a transforaminal injection is given at the eye of the Scottie dog, which is obtained by moving the C-arm 20-30 degrees obliquely ipsilaterally. I/L: Ipsilateral, C/L: Contralateral, IAP: Inferior articular process, SAP: Superior articular process, TP: Transverse process.

**Figure 3:**
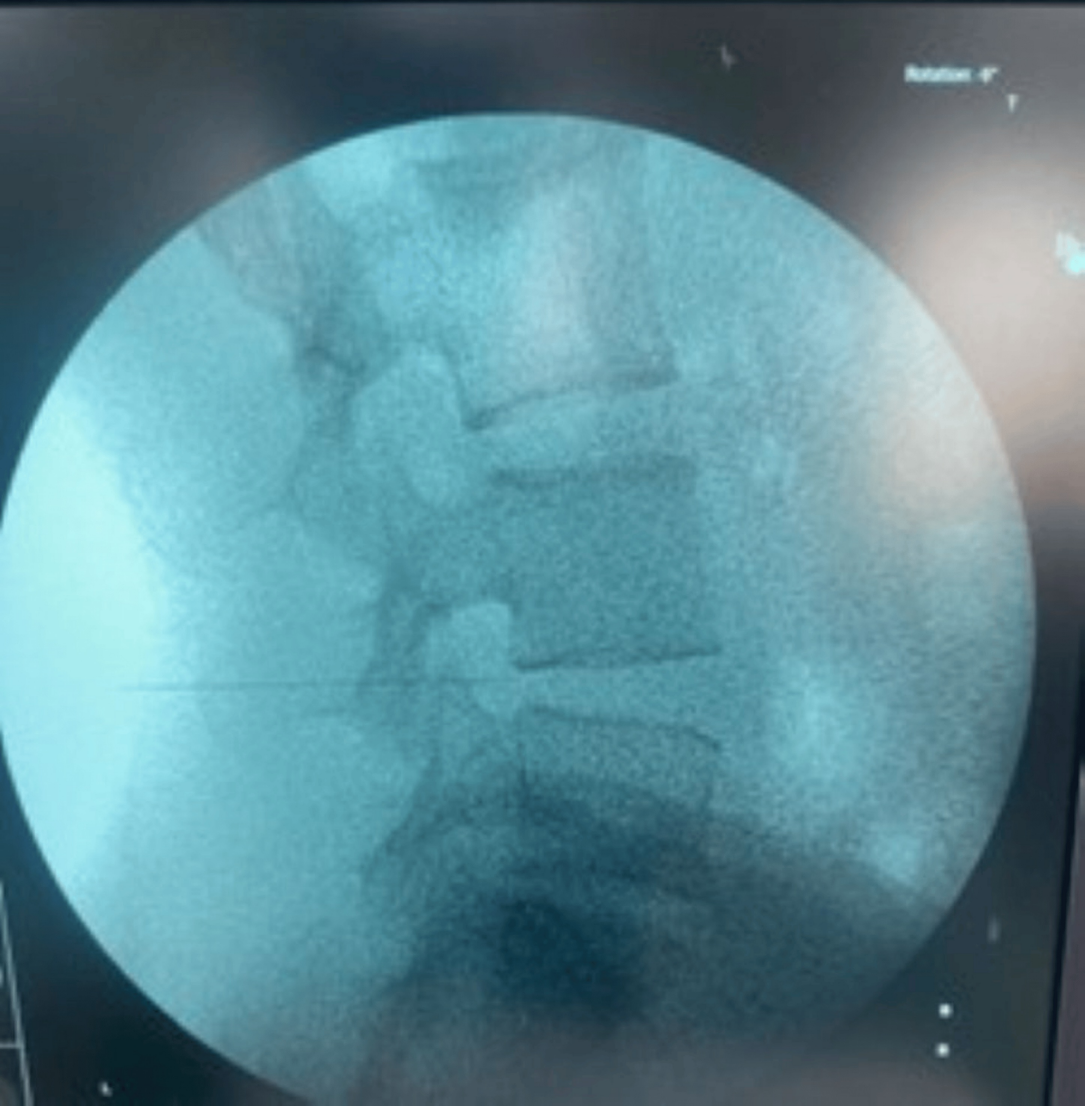
Depth of the needle checked under lateral view

**Figure 4:**
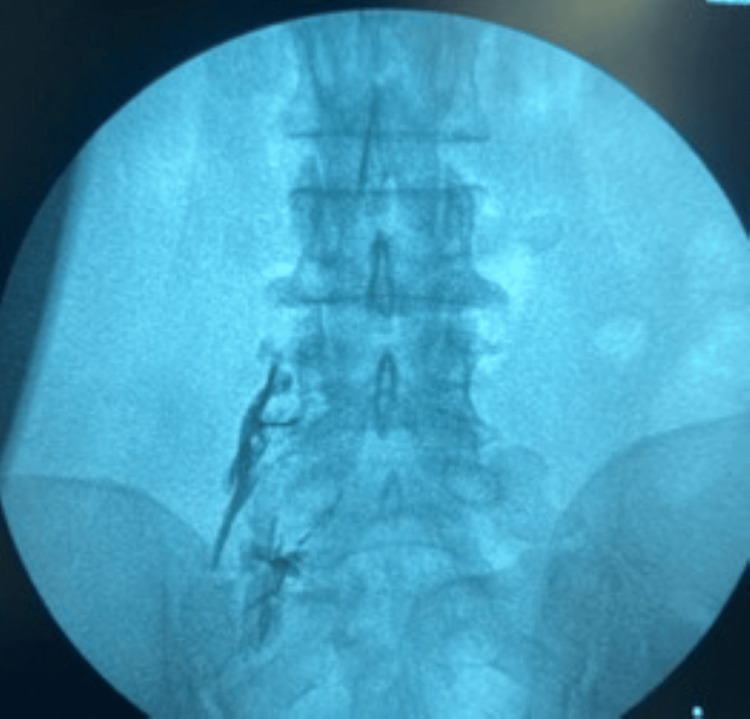
Nerve root pattern after giving contrast

Based on the treatment received as part of routine clinical management, patients were classified into two observational cohorts. Patients who underwent TFESI as part of standard care received the procedure under strict aseptic precautions in the operating theatre with the patient in the prone position. After skin preparation and local infiltration with 2% lidocaine, the target level was identified under fluoroscopic guidance. Using oblique and lateral C-arm views, a 22-gauge spinal needle was advanced into the lateral epidural space through the intervertebral foramen. Correct needle placement was confirmed by injection of non-ionic contrast medium, demonstrating appropriate nerve root spread. In patients with two-level disc involvement, the clinically dominant symptomatic level was selected for injection. Subsequently, 40 mg of triamcinolone acetonide combined with 1 mL of 2% lidocaine was administered. Patients were observed post-procedure for any immediate complications (Figures 2-4).

Patients managed with oral corticosteroids as part of routine treatment received oral prednisolone for a total duration of 15 days in a tapering regimen: 60 mg once daily for the first five days, followed by 40 mg once daily for the next five days, and 20 mg once daily for the final five days, resulting in a cumulative dose of 600 mg. This regimen was based on the protocol described by Goldberg et al. [17]. Dose modification was permitted only in the event of adverse effects. No attempt was made by the investigators to influence the choice of treatment modality. Rescue analgesia with paracetamol (up to 3 g/day) was permitted in both groups. NSAIDs were discontinued at the time of steroid initiation. Neuropathic agents (gabapentinoids or tricyclic antidepressants) were not initiated during the first six weeks of follow-up. Structured physiotherapy was deferred during the initial six weeks and permitted thereafter if clinically indicated.

Patients were evaluated at baseline and at 1, 3, 6, and 12 weeks following initiation of treatment. Pain intensity, functional disability, and nerve root tension were assessed at each follow-up visit using standardized outcome measures. Pain intensity was measured using the Visual Analogue Scale (VAS), a 10-cm scale where 0 indicates no pain and 10 indicates the worst imaginable pain [18].

Functional disability was assessed using the Modified Oswestry Disability Index (MODI), expressed as a percentage score. The MODI used in this study was adapted from the original Oswestry Disability Index developed by Fairbank and Pynsent and was not developed by the authors of this study [19]. The questionnaire consists of 10 sections, each scored from 0 to 5. The total score was calculated by summing the scores of all sections and converting it to a percentage using the formula: (Total score/50) × 100. Interpretation of MODI scores was as follows: 0-20% = minimal disability; 21-40% = moderate disability; 41-60% = severe disability; 61-80% = crippled; and 81-100% = bed-bound or exaggerating symptoms. For statistical analysis, MODI scores were treated as continuous variables and expressed as mean ± standard deviation (SD).

Nerve root tension was evaluated using the Straight Leg Raise Test (SLRT), measured in degrees [20]. SLRT was interpreted as positive when reproduction of radicular leg pain occurred between 30° and 70° of hip flexion and was expressed as mean ± standard deviation (SD). 

Outcome assessments were performed by an orthopedic resident who was not involved in treatment allocation or procedural execution to minimize observer bias. Minor and major adverse events were actively monitored and documented at each follow-up visit.

Data collected included demographic variables, clinical findings, VAS scores for back and leg pain, MODI scores, SLRT measurements, MRI characteristics (level and type of disc herniation), procedural duration, level of injection, and contrast dye diffusion pattern. Statistical analysis was performed using MedCalc software (version 20.019). Normality of data distribution was assessed using the Shapiro-Wilk test. Parametric data were expressed as mean ± standard deviation, while non-parametric data were expressed as median with interquartile range. Between-group comparisons were performed using the independent t-test or Mann-Whitney U test, while within-group comparisons were conducted using the paired t-test or Wilcoxon signed-rank test, as appropriate. Categorical variables were analyzed using the chi-square test. A p-value of less than 0.05 was considered statistically significant.

The study was conducted in accordance with the Declaration of Helsinki. Ethical approval was obtained from the Institutional Ethics Committee, AIIMS Bibinagar (IEC No: AIIMS/BBN/IEC/MAR/2023/271-R). Written informed consent was obtained from all participants prior to enrolment, and confidentiality of patient data was strictly maintained.

## Results

A total of 34 patients were observed, 17 in each group. Baseline demographic and clinical characteristics were comparable between the TFESI and oral steroid groups. There were no significant differences in age distribution, gender, occupation, baseline pain intensity (VAS for back and leg pain), functional disability (MODI), or nerve root tension (SLRT), indicating similar baseline clinical status prior to intervention (all p > 0.05) (Table 1).

**Table 1:** Demographic and clinical characteristics at baseline. Values are expressed as mean ± SD, median (IQR), or number (%), as appropriate. Between-group comparisons were performed using the independent t-test (age, MODI, SLRT), Mann–Whitney U test (VAS back and leg pain), and chi-square test (categorical variables). IQR: interquartile range; MODI: Modified Oswestry Disability Index; SLRT: Straight Leg Raise Test.

Characteristic		TFESI (n=17)	Oral Steroid (n=17)	Test statistic	p-value
Age (years)	Mean ± SD	44.59 ± 9.06	42.00 ± 9.00	t = 0.86	0.394
Age categories	18-30 years	2 (11.8)	3 (17.6)	χ² = 0.562	0.967
	31-40 years	3 (17.6)	4 (23.5)		
	41-50 years	7 (41.2)	6 (35.3)		
	51-60 years	4 (23.5)	3 (17.6)		
	61-70 years	1 (5.9)	1 (5.9)		
Gender	Male	9 (52.9)	8 (47.1)	χ² = 0.12	0.732
	Female	8 (47.1)	9 (52.9)		
Occupation	Farmer	4 (23.5)	4 (23.5)	NA	NA
	Labourer	3 (17.6)	3 (17.6)		
	Housewife	6 (35.3)	6 (35.3)		
	Private job	4 (23.5)	4 (23.5)		
VAS back pain	median (IQR)	5.0 (4.0-6.0)	5.0 (5.0-6.0)	U = 106	0.168
VAS leg pain	median (IQR)	7.0 (6.0-7.5)	7.0 (7.0-8.0)	U = 134	0.635
MODI score (%)	mean ± SD (95% CI)	60.47 ± 6.10 (57.32-63.62)	50.59 ± 5.42 (47.79-53.39)	t = 1.74	0.092
SLRT (degrees)	mean ± SD (95% CI)	62.94 ± 14.90 (55.27-70.61)	69.00 ± 14.00 (61.80-76.20)	t = –1.28	0.208

MRI characteristics did not differ significantly between groups with respect to level of involvement, pathology type, or side of radiculopathy. Most patients had single- or two-level disease, predominantly at the L4-L5 level, with disc bulge or protrusion as the most common pathology and comparable frequencies of associated degenerative findings across groups (Table 2).

**Table 2:** MRI findings (etiology of low back pain) Data are presented as number (%). Between-group comparisons were performed using the Chi-square test. MRI, magnetic resonance imaging.

Characteristic		TFESI (n=17)	Oral Steroid (n=17)	χ² value	p-value
Level of involvement	L4-L5 only	8 (47.1)	7 (41.2)	0.01	0.999
	L5-S1 only	1 (5.9)	1 (5.9)		
	L4-L5 and L5-S1	4 (23.5)	5 (29.4)		
	L3-L4 and L4-L5	2 (11.8)	2 (11.8)		
	Multilevel (≥3 levels)	2 (11.8)	2 (11.8)		
Pathology type	Disc bulge	9 (52.9)	10 (58.8)	0.44	0.804
	Disc protrusion	7 (41.2)	6 (35.3)		
	Stable spondylolisthesis	1 (5.9)	1 (5.9)		
Side of radiculopathy	Left	10 (58.8)	11 (64.7)	0.10	0.749
	Right	7 (41.2)	6 (35.3)		
Additional findings	Neural foraminal stenosis	5 (29.4)	6 (35.3)	0.134	0.717
	Ligamentum flavum hypertrophy	2 (11.8)	1 (5.9)	0.365	0.547
	Disc desiccation	4 (23.5)	3 (17.6)	0.179	0.677

Between-group comparison of pain scores demonstrated significantly lower VAS back and leg pain in the TFESI group from 1 to 6 weeks compared with the oral steroid group, with attenuation of this difference by 12 weeks. No significant differences were observed at baseline for either pain measure (Table 3).

**Table 3:** Between-group comparison of VAS scores over time (median [IQR]) Values are expressed as medians (IQR). Between-group comparisons were performed using the Mann–Whitney U test. VAS: Visual Analogue Scale; IQR: interquartile range.

Parameter	Time point	TFESI (n=17)	Oral Steroid (n=17)	U-value	p-value
VAS Back Pain	Baseline	5.0 (4.0–6.0)	5.0 (5.0–6.0)	106	0.168
	1 week	2.0 (1.0–2.0)	5.0 (4.0–5.5)	4	<0.001
	3 weeks	2.0 (1.5–2.5)	5.0 (4.0–5.0)	6	<0.001
	6 weeks	3.0 (2.0–3.0)	4.0 (4.0–5.0)	12	<0.001
	12 weeks	3.0 (3.0–4.0)	4.0 (3.5–4.5)	91	0.064
VAS Leg Pain	Baseline	7.0 (6.0–7.5)	7.0 (7.0–8.0)	134	0.635
	1 week	2.0 (2.0–2.5)	6.0 (5.0–7.0)	3	<0.001
	3 weeks	2.0 (2.0–3.0)	5.0 (4.0–5.0)	7	<0.001
	6 weeks	3.0 (3.0–3.5)	5.0 (4.0–5.0)	9	<0.001
	12 weeks	4.0 (3.0–4.0)	5.0 (4.0–5.0)	88	0.058

Within-group analysis showed significant early reductions in both back and leg pain in the TFESI group, particularly from baseline to 1 week and from 3 to 6 weeks, whereas improvements in the oral steroid group were smaller and less consistent over time, with fewer statistically significant interval changes (Table 4).

**Table 4:** Within-group comparison of VAS scores over time The data represent changes in pain scores within each treatment group across follow-up intervals. Comparisons were performed using the Wilcoxon signed-rank test. VAS: Visual Analog Scale.

Parameter	TFESI		Oral Steroid	
	Z-value	p-value	Z-value	p-value
VAS Back Pain				
Baseline vs. 1 week	–3.62	<0.001	–1.14	0.253
1 vs. 3 weeks	–0.50	0.614	–0.54	0.587
3 vs. 6 weeks	–2.75	0.006	–2.36	0.018
6 vs. 12 weeks	–2.86	0.004	–1.13	0.260
VAS Leg Pain				
Baseline vs 1 week	–3.62	<0.001	–1.25	0.212
1 vs. 3 weeks	–1.65	0.098	–1.80	0.072
3 vs. 6 weeks	–2.43	0.015	–0.40	0.687
6 vs. 12 weeks	–2.95	0.003	–0.20	0.842

Between-group comparison of functional outcomes revealed significantly greater improvement in MODI scores and SLRT angles in the TFESI group at early follow-up (1 and 3 weeks), with sustained differences up to 6 weeks for disability scores. By 12 weeks, between-group differences diminished and were no longer statistically significant (Table 5).

**Table 5:** Between-group comparison of MODI score and SLRT over time Values are expressed as mean ± SD (95% confidence interval). Between-group comparisons were performed using the independent t-test. MODI: Modified Oswestry Disability Index; SLRT: Straight Leg Raise Test.

Parameter	Time point	TFESI (n=17)	Oral Steroid (n=17)	t-value	p-value
MODI Score (%)	Baseline	60.47 ± 6.10 (57.32–63.62)	50.59 ± 5.42 (47.79–53.39)	1.74	0.092
	1 week	24.00 ± 3.08 (22.41–25.59)	39.76 ± 6.96 (36.17–43.35)	5.12	<0.001
	3 weeks	23.88 ± 2.18 (22.76–25.00)	37.29 ± 5.24 (34.58–40.00)	5.46	<0.001
	6 weeks	26.59 ± 3.14 (24.96–28.22)	36.82 ± 4.64 (34.42–39.22)	4.91	<0.001
	12 weeks	30.59 ± 3.52 (28.77–32.41)	35.18 ± 5.39 (32.39–37.97)	1.95	0.057
SLRT (degrees)	Baseline	62.94 ± 14.90 (55.27–70.61)	69.00 ± 14.00 (61.80–76.20)	1.28	0.208
	1 week	82.94 ± 5.88 (79.91–85.97)	70.00 ± 11.00 (64.34–75.66)	4.67	<0.001
	3 weeks	77.65 ± 5.62 (74.75–80.55)	73.00 ± 8.00 (68.88–77.12)	1.82	0.076
	6 weeks	72.94 ± 7.72 (68.95–76.93)	72.00 ± 7.00 (68.39–75.61)	0.39	0.695
	12 weeks	74.00 ± 7.00 (70.39–77.61)	74.00 ± 9.00 (69.38–78.62)	0.73	0.471

Within-group analysis of MODI and SLRT demonstrated marked early improvement in the TFESI group, particularly from baseline to one week, with partial decline at later follow-up. In contrast, the oral steroid group showed more gradual and modest improvement, with fewer significant interval changes (Table 6).

**Table 6:** Within-group comparison of MODI score and SLRT over time Data represents interval changes within each treatment group across follow-up time points. Comparisons were performed using the paired t-test. MODI: Modified Oswestry Disability Index; SLRT: Straight Leg Raise Test.

Parameter	TFESI		Oral Steroid	
	t-value	p-value	t-value	p-value
MODI Score (%)				
Baseline vs. 1 week	6.21	<0.001	5.47	<0.001
1 vs. 3 weeks	0.16	0.875	2.29	0.037
3 vs. 6 weeks	3.32	0.003	0.61	0.547
6 vs. 12 weeks	5.04	<0.001	2.24	0.034
SLRT (degrees)				
Baseline vs. 1 week	5.86	<0.001	1.38	0.182
1 vs. 3 weeks	3.18	0.003	2.44	0.018
3 vs. 6 weeks	2.27	0.031	0.89	0.385
6 vs. 12 weeks	0.82	0.427	1.64	0.120

At 12 weeks, a higher proportion of patients in the TFESI group achieved more than 50% improvement in VAS back pain, VAS leg pain, and MODI scores compared with the oral steroid group; however, these differences did not reach statistical significance (Table 7).

**Table 7:** Patients with more than 50% improvement at 12 weeks Values are expressed as number (%). Between-group comparisons were performed using the Chi-square test. VAS: Visual Analogue Scale; MODI: Modified Oswestry Disability Index.

Parameter	Time point	TFESI (n = 17) N (%)	Oral Steroid (n = 17) N (%)	χ² value	p-value
VAS Back Pain	12 weeks	5 (29.4%)	2 (11.8%)	1.70	0.193
VAS Leg Pain	12 weeks	8 (47.1%)	3 (17.6%)	3.35	0.068
MODI Score	12 weeks	9 (52.9%)	2 (11.8%)	3.45	0.064

Procedure-related parameters in the TFESI group indicated a short and consistent procedure duration, with most injections administered at the L4-L5 level. Multilevel injections were infrequent, and intraoperative adverse findings were rare, underscoring the procedural feasibility and uniformity of TFESI in this cohort (Table 8).

**Table 8:** TFESI procedure parameters (n=17) Values are expressed as mean ± SD (95% confidence interval), range, or number (%), as appropriate. Procedure time is presented as mean duration with the observed minimum and maximum values (range). TFESI: transforaminal epidural steroid injection; CI: confidence interval; min: minutes.

Parameter		Value
Procedure time	mean ± SD (95% CI)	15.53 ± 1.84 min (range: 12–18 min) 95% CI: 14.58-16.48
Level of injection	L4-L5	13 (76.5)
	L5-S1	1 (5.9)
	Multiple levels	3 (17.6)
Intraoperative findings	Narrowed intervertebral foramen	1 (5.9)
	None	16 (94.1)

No major or minor adverse events were observed in either treatment group during the 12-week follow-up period. No patient required treatment discontinuation, hospitalization, or additional intervention related to the study treatments.

## Discussion

Acute lumbar radiculopathy constitutes a major source of disability and economic burden, particularly among working-age adults, with direct and indirect costs rivaling those of chronic systemic diseases [21]. In the present prospective comparative analysis, both treatment groups were comparable at baseline with no statistically significant differences with respect to age, sex distribution, pain intensity, functional disability, neural tension, and radiological characteristics, thereby minimizing confounding and allowing meaningful comparison of post-intervention outcomes [4,22,23]. The predominance of middle-aged individuals and L4-L5 disc involvement observed in this cohort mirrors the epidemiological profile reported in previous large series, reinforcing the external validity of the findings [24].

A consistent and clinically important observation was the marked early association of TFESI with greater improvement compared to oral corticosteroids across pain, disability, and objective neural tension measures. Within the first week, TFESI resulted in rapid and substantial reductions in both back and leg pain, with median improvements far exceeding those achieved by oral prednisolone. Similar early benefits have been reported by Ghahreman et al. and Vad et al., who attributed this effect to direct ventral epidural delivery of corticosteroids adjacent to the affected nerve root, resulting in prompt suppression of inflammatory mediators and periradicular edema [22,25]. The more pronounced improvement in radicular pain relative to axial pain further supports the mechanistic specificity of the transforaminal approach, as previously proposed by Kennedy et al. [24].

Functional recovery, as assessed by the Modified Oswestry Disability Index, closely paralleled pain relief patterns. The TFESI group demonstrated a steep early decline in disability scores, with improvements exceeding the minimum clinically important difference by more than threefold within the first week. These early gains are comparable to those reported in interventional trials and approach the magnitude of improvement described following surgical decompression in selected cohorts [26,27]. Although oral corticosteroids also produced measurable functional improvement, the onset was delayed and the magnitude consistently inferior during the first six weeks. This divergence suggests that early suppression of periradicular inflammation may play a key role in restoring mobility and reducing activity limitation.

Objective improvement in nerve root tension, reflected by increases in Straight Leg Raise Test (SLRT) values, provides biological corroboration of subjective pain and disability outcomes. Rapid SLRT improvement in the TFESI group has also been documented by Friedly et al., who suggested that early resolution of periradicular inflammation rather than structural disc regression accounts for this phenomenon [23]. In the present study, SLRT values in the TFESI group approached functional normalization during early follow-up, while systemic corticosteroids produced slower and less pronounced changes in neural tension, consistent with their indirect and diffuse mechanism of action.

Despite clear early differences, the progressive convergence of outcomes between groups by 12 weeks aligns with established pharmacokinetic and natural-history data. Depot corticosteroids such as triamcinolone acetonide exert their maximal anti-inflammatory effect over a limited period of approximately 2-4 weeks, after which attenuation of clinical benefit may occur due to declining local drug concentration [28]. Concurrently, spontaneous resorption of herniated disc material and gradual resolution of nerve root inflammation have been well documented, contributing to clinical improvement irrespective of treatment modality [29]. Similar convergence of pain and functional outcomes over time has been observed in randomized trials evaluating epidural steroid injections [23].

These findings have practical clinical implications. TFESI may be particularly beneficial in patients requiring rapid symptom control to facilitate early mobilization, return to work, or participation in rehabilitation programs. In contrast, oral corticosteroids may represent a reasonable non-invasive alternative for patients preferring conservative escalation and who can tolerate a slower trajectory of symptom improvement.

From a safety perspective, transforaminal epidural steroid injection (TFESI) demonstrated a favorable procedural profile with minimal technical challenges and limited systemic steroid exposure. Importantly, no major or minor adverse events were observed in either group during the 12-week follow-up period. While short courses of oral corticosteroids are associated with potential metabolic, gastrointestinal, and endocrine effects [30], such events were not observed in the present cohort. Nevertheless, the modest sample size limits the ability to detect rare adverse events.

Strengths and limitations

The strengths of the present study include its prospective observational design, which allowed systematic follow-up and temporal assessment of outcomes after initiation of routine clinical treatment. The use of a standardized corticosteroid molecule across both treatment modalities reduced pharmacological variability and enhanced comparability between groups. Strict imaging-based eligibility criteria ensured diagnostic homogeneity, and repeated outcome assessment at multiple time points enabled evaluation of early and short-term response patterns. These methodological features strengthen internal consistency and support meaningful temporal interpretation of treatment-associated changes.

Several limitations should be acknowledged. First, the absence of randomization introduces potential selection bias, as treatment allocation was determined by clinical judgment and patient preference. Second, the relatively small sample size (n = 34) limits statistical power, particularly for secondary outcomes and safety analysis, and restricts generalizability. Third, the open-label nature of the study may have influenced patient-reported outcomes through expectancy effects. Fourth, the follow-up duration of 12 weeks precludes conclusions regarding long-term durability of symptom relief, recurrence rates, need for surgical intervention, and cost-effectiveness. Accordingly, the findings should be interpreted as preliminary observational evidence rather than definitive proof of treatment superiority. Larger, multicenter randomized controlled trials with extended follow-up are required to validate these results and to better define optimal patient selection criteria.

## Conclusions

The present study suggests that, in this prospective non-randomized observational cohort, a single fluoroscopy-guided TFESI using triamcinolone acetonide was associated with faster and more pronounced short-term pain relief and functional improvement compared with oral corticosteroid therapy in adults with acute lumbar radiculopathy refractory to initial conservative management. TFESI was associated with substantial early reductions in both leg and back pain, along with marked improvement in functional disability and objective neural tension during the first six weeks of follow-up. Although outcome differences narrowed by 12 weeks, reflecting the waning depot steroid effect and the influence of natural disease resolution, TFESI demonstrated a clinically meaningful early advantage within the study period with no observed adverse events. These findings indicate that TFESI may represent a valuable early interventional option for appropriately selected patients with MRI-confirmed discogenic radiculopathy who require rapid symptom relief. Larger randomized controlled trials with extended follow-up are warranted to confirm these observations and to clarify long-term durability, cost-effectiveness, and optimal integration with rehabilitation strategies.
